# Anticodon Wobble Uridine Modification by Elongator at the Crossroad of Cell Signaling, Differentiation, and Diseases

**DOI:** 10.3390/epigenomes4020007

**Published:** 2020-05-12

**Authors:** Damien Hermand

**Affiliations:** URPHYM-GEMO, The University of Namur, 5000 Namur, Belgium; Damien.Hermand@unamur.be

**Keywords:** tRNA, modification, anticodon, yeast, wobble

## Abstract

First identified 20 years ago as an RNA polymerase II-associated putative histone acetyltransferase, the conserved Elongator complex has since been recognized as the central player of a complex, regulated, and biologically relevant epitranscriptomic pathway targeting the wobble uridine of some tRNAs. Numerous studies have contributed to three emerging concepts resulting from anticodon modification by Elongator: the codon-specific control of translation, the ability of reprogramming translation in various physiological or pathological contexts, and the maintenance of proteome integrity by counteracting protein aggregation. These three aspects of tRNA modification by Elongator constitute a new layer of regulation that fundamentally contributes to gene expression and are now recognized as being critically involved in various human diseases.

## 1. Introduction

As a model organism, yeast has contributed enormously to most, if not all, aspects of our understanding of the fundamental biology of eukaryotes, ranging from cell cycle, cytoskeleton, autophagy, chromosome biology, metabolism, and gene expression. In that context, the *Saccharomyces cerevisiae* (*S. cerevisiae*, budding yeast) Elongator complex was found to be associated with the CTD (C-terminal domain) hyperphosphorylated form of RNA polymerase II (Pol II), which was already known by that time to represent the elongating version of the Pol II complex [1]. The Elongator was first reported to include three proteins, Elp1, Elp2, and Elp3, but subsequently shown to also harbor the Elp4, Elp5, and Elp6 subcomplexes [2,3]. These findings already suggested the architectural features of a holo-Elongator, which was confirmed and expanded upon later. The Elongator was first co-purified with transcribing Pol II [4,5]. In addition, the complex displayed detectable histone H3 and histone H4 lysine acetyltransferase activity, and the catalytic subunit Elp3 contained a domain homologous to that found in members of the GNAT (Gcn5-related N-terminal acetyltransferase) superfamily. Taken together, these data suggested a direct, supporting role in transcription through the chromatin template [4,5]. The sensitivity of Elongator mutants to 6-azauracil (a drug that affects transcriptional elongation), the synthetic lethality between Elongator mutants and alleles of the gene encoding Gcn5 histone acetyltransferase (HAT), and the delayed ability of strains lacking Elongator to induce gene expression reinforced a model where Elongator accompanies Pol II and participates in the production of mRNAs [3,6]. Nevertheless, while Elongator was later shown to bind nascent mRNAs [7], another study reported that it did not purify with well-described Pol II associated factors [8]. Most puzzling, strong evidence was reported that one of the phenotypes of a yeast Elongator mutant, a disrupted Sec2 localization and consequent inefficient exocytosis, resulted from a defect occurring outside of the nucleus [9]. These data, inconsistent with the proposed model, raised doubts about it, which were in turn nuanced by the possibility that Elongator may take on different functions and roles through the modification of a range of substrates [10]. Indeed, a possible different nuclear function of Elongator in telomeric gene silencing and DNA repair was also reported [11]. In addition to the HAT domain mentioned above, the Elp3 subunit possesses another region sharing homology to proteins from the radical S-adenosylmethionine (SAM) family containing an iron-sulfur cluster [12]. It has been reported that Elongator has a role in zygotic paternal genome demethylation that exclusively requires the SAM domain [13]. As shown above, proponents of either a model where a unique substrate affects several biological processes, or an alternative model where Elongator affects multiple substrates resulting in pleiotropic phenotypes, all have had arguments to make. A very elegant genetic approach has helped to clarify this issue [14].

**Figure 1:**
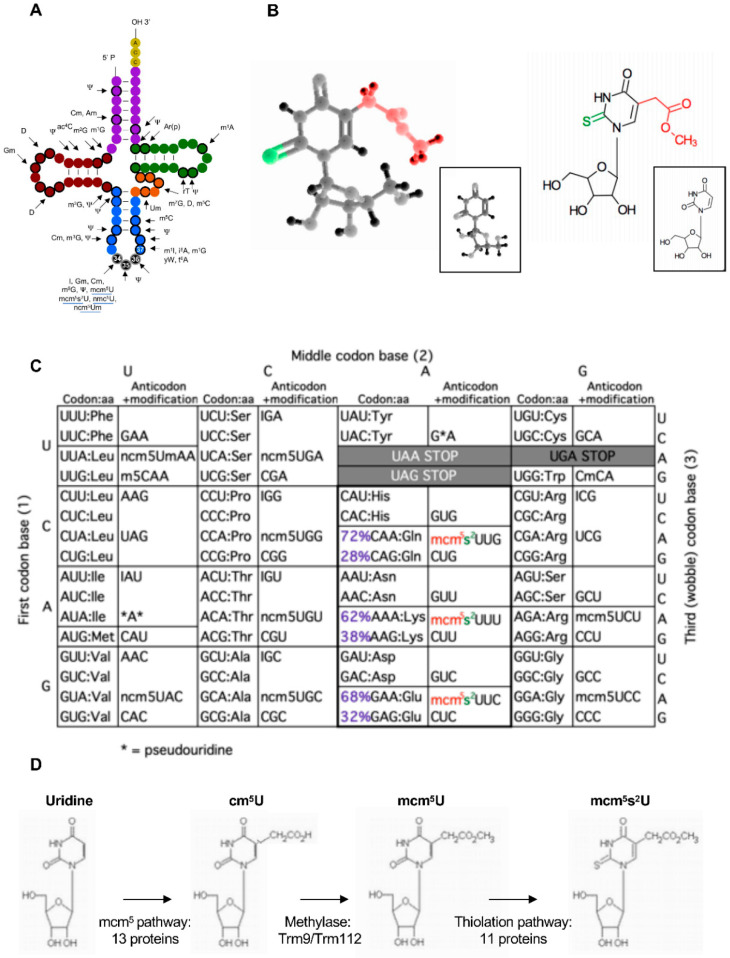
Elongator-dependent modifications of tRNAs. (**A**) tRNA modifications found in cytoplasmic tRNAs of *S. cerevisiae*. Bold circles indicate modified positions in some or all tRNAs. Black circles represent the anticodon corresponding to positions 34, 35, and 36. The highly modified position 37 is also indicated. The Elongator-dependent modifications are underlined (modified from Phizicky and Hopper, 2010 [15]). (**B**) Tri- (left) or bidimensional (right) models of uridine (insert) and its doubly modified mcm^5^s^2^ version. (**C**)The genetic code with codons, anticodons, and the elongator-dependent modifications, ncm^5^, mcm^5^, and mcm^5^s^2^ found on 11 tRNAs. Note that the mcm^5^s^2^-modified tRNAs recognize codons in the split boxes. For glutamine (Gln), lysine (Lys), and glutamic acid (Glu), the codon usage of *S. pombe* is indicated. The A-ended codon is favored due to the low G-C content of the fission yeast genome. “I” stands for inosine and the asterisk for pseudouridine. (**D**) Steps in the synthesis of the mcm^5^s^2^ modification and the number of required proteins. The six subunits of Elongator are included in the 13 proteins required to synthetize cm^5^U. Trm12 is a physical partner of the Trm9 methylase.

**Figure 2:**
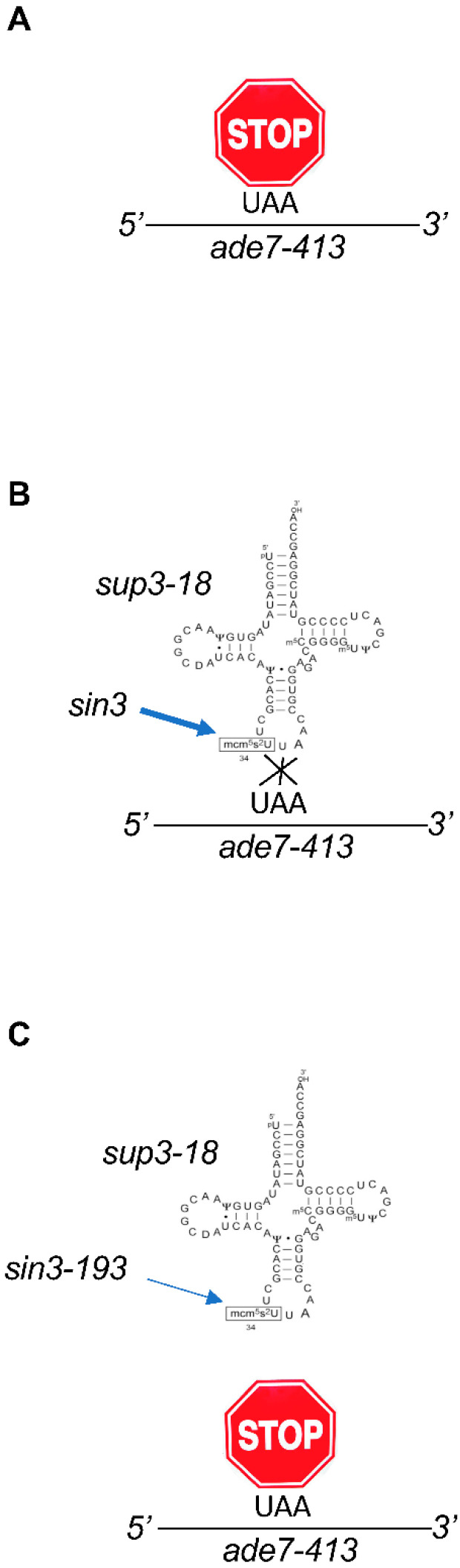
Identification of the antisuppressor *sin3-193* mutant as an allele of *elp3* in fission yeast. (**A**) The *ade7-413* allele of the fission yeast *ade7* gene contains an ochre STOP codon (UAA). The mutation results in adenine auxotrophy and red color (not shown). (**B**) The suppressor tRNA *sup3-18* contains an anticodon mutation able to read the UAA STOP codon and incorporate a serine, which suppresses adenine auxotrophy and red color (not shown). The *sin3* (Elp3) gene product is required for both the anticodon modification (mcm^5^s^2^) and efficient suppression. Note the consistent 5′-3′polarity of the tRNA and mRNA molecules, resulting in the reverse orientation of the codon and the anticodon. (**C**) The *sin3-193* allele of *sin3* (Elp3) is a loss-of-function allele and results in anti-suppression. Therefore, despite the presence of the suppressor tRNA, the strain is red and an auxotroph for adenine (not shown), which are easily followed phenotypes.

tRNAs from all organisms contain modified nucleosides [15], and when a uridine is present in the wobble position (U_34_), this residue is almost universally modified (Figure 1) [16,17,18]. Specifically, in eukaryotes, the tRNAs reading codons belonging to split codon boxes, which include the tRNA^Lys^_UUU_, tRNA^Glu^_UUC_, and tRNA^Gln^_UUG_, are thiolated (s^2^) at the 2-carbon and contain a methoxy-carbonyl-methyl modification (mcm^5^) at the 5-carbon on the uridine. This complex double modification (mcm^5^s^2^U_34_) is required to offset the translational inefficiency of the AA-ending codons (XAA) within the corresponding two-codon boxes [19,20]. The complete thiolation pathway has been described with Ctu1 catalyzing the final step [21,22,23]. Some tRNAs also bear only the mcm^5^ moiety or a related modification called ncm^5^ (for carbamoylmethyluridine). In budding yeast, where this is best known, there are 42 cytosolic tRNAs species and 11 of these contain ncm^5^U (6 tRNAs), mcm^5^U (2 tRNAs), or mcm^5^s^2^U (3 tRNAs) (Figure 1) [24,25]. This prevalence and the intriguing complexity of these modifications motivated efforts for the identification of the enzymes and other proteins required to establish them, which was challenging in the absence of tools to detect them. However, a mutant of *Schizosaccharomyces pombe* (*S. pombe*, a fission yeast only distantly related to budding yeast) called *sin3-193* was isolated in the eighties and shown to display various phenotypes, including discrete cell cycle defects [26,27]. Interestingly, the *sin3* mutant was shown by liquid chromatographic analysis to have a very low level of the mcm^5^s^2^U nucleoside, which suggested that the mutation likely occurred in a gene required for the synthesis of this modification. The low level of the modification was correlated to an anti-suppressor phenotype [28]. In simple terms, this means that a mutated tRNA whose anticodon can read a STOP codon (called a suppressor tRNA) requires the modification to be functional and bypass the presence of a STOP within a given mRNA (Figure 2).

**Figure 3:**
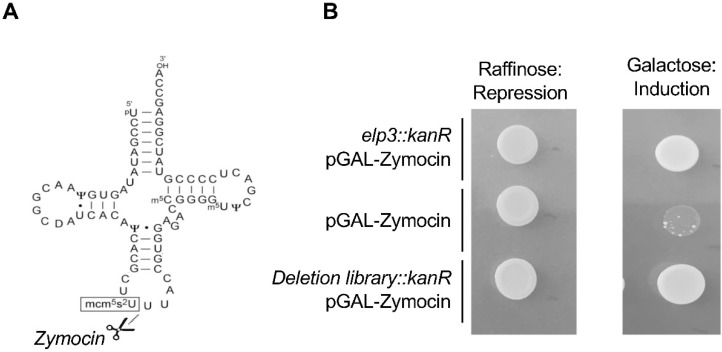
Identification of the genes necessary for the synthesis of the mcm^5^s^2^ modification in budding yeast using zymocin. (**A**) Zymocin is a tRNA endonuclease that cleaves the anticodon of tRNAs bearing the mcm^5^s^2^ modification. (**B**) Screening set-up. The zymocin-encoding gene is expressed from the *GAL* promoter that is repressed by raffinose and induced by galactose. The growth of a wild-type strain on galactose (middle) results in death, due to the cleavage of the target tRNAs. An Elongator mutant (*elp3::kanR*) is resistant to zymocin due to the absence of this modification. Expression of zymocin in the yeast deletion library (a collection of yeast strains where all viable haploid strains deleted for a given gene are represented) allows the identification of all the non-essential genes required for the mcm^5^s^2^ modification, which was confirmed by HPLC analysis of tRNAs.

The team of Anders Byström used complementation to identify the *sin3* gene, which turned out to be the fission yeast orthologue of Elp3 [29] that encodes the catalytic subunit of Elongator. What is remarkable about this work is that it already encompasses some of the later key findings related to Elongator, namely that the complex is required for the synthesis of the mcm^5^ modification, which is itself required for efficient decoding of mRNAs with discrete phenotypical implications rather than a general unspecific defect. Next, in a series of remarkable works, the same team quickly identified all the actors of the pathway leading to the complex double mcm^5^s^2^ modifications and also the single mcm^5^ and ncm^5^ variants [30]. A key finding allowing this genetic work was the discovery that tRNAs bearing the Elongator-dependent modification are the primary targets of zymocin, an endonuclease toxin recognizing the mcm^5^ modification to cut the anticodon of the corresponding tRNAs and kill the cell [31]. All yeast strains with reduced levels, or in the absence of the mcm^5^s^2^ modification, are resistant to endogenous expression of the toxin (so-called Type II mutants in opposition to Type I mutants that do not allow zymocin to enter the cell), allowing the screening of the yeast deletion library (Figure 3). Besides all six Elongator subunits, this led to the identification of Trm9 as the enzyme required for the terminal methylation of the mcm^5^ modification. Another set of proteins identified in the screen was suggested to regulate the activity of Elongator and the level of the modification, an unexpected possibility at a time where tRNA modifications were considered to be stable and constitutive. These proteins include the Kti11-14 proteins, Sit4, and the Sap185/Sap190 proteins. Sit4 is a type 2A phosphatase that requires members of the Sap group as effectors or activators [32]. Kti14 encodes a kinase also named Hrr25, a homologue of the mammalian casein kinase 1δ, suggesting the intriguing possibility that both a kinase and a phosphatase are equally needed for Elongator activity (see below). Kti11 and Kti13 form a complex that was later shown to be implicated in electron transfer to Elp3 for the radical SAM-dependent activity mentioned above [33,34].

The discovery that Elongator is critical for mcm^5^/ncm^5^ tRNA modification allowed the Byström team to test if this could be the genuine function of the complex. In a milestone paper from 2006 [35], they showed that all the reported defects resulting from the inactivation of Elongator in yeast, except the tRNA modification itself, could be suppressed by overexpression of the unmodified tRNA^Lys^_UUU_ and to a lesser extend tRNA^Glu^_UUC_. The overexpression of any of the other 9 target tRNAs did not, pointing to a more specific requirement of the modification to properly decode the AAA codon and possibly the GAA codon. Further supporting the already strong genetic evidence, these defects were all observed, albeit often to a lower extent when the thiolation (s^2^), which requires a totally independent pathway, was inactivated. Therefore, in budding yeast, Elongator functions as a tRNA modification enzyme and its complete inactivation rubs off on various cellular pathways, ranging from transcriptional induction to secretion or telomere maintenance. 

At this stage however, many questions remained unanswered, for example, why is the modification pathway so complex? Which mRNAs, if any, are specifically sensitive to modification during translation? Is this pathway counteracting weak codon-anticodon interactions constitutive or is it integrated with other cellular signaling cascades? In addition, the mechanistic details of the synthesis of the modification and the structure/function relationship of the complex were also completely unknown.

## 2. Codon-Based Regulation of Translation

Another milestone study was published by the team of Thomas Begley in 2007. Although not directly focusing on Elongator, the work described how the Trm9 methylase that completes the cm^5^ modification of tRNAs by Elongator links translation to the DNA damage response [36]. An original proposal of this work is that the skewed codon content of some mRNAs makes them very sensitive to a specific modification. In addition, these mRNAs may belong to a functional group, therefore leading to a novel mechanism of coordinated, codon-based regulation of transcription. The development of a computational approach to compile and visualize gene-specific codon usage for all budding yeast genes as hierarchical clusters displayed as heat maps revealed an unexpected feature. Indeed, a group of genes clustered together with a pronounced skewed codon content, especially for the AGA codon, at the expense of the synonymous AGG codon. Interestingly, Trm9 and Elongator cooperate to generate the mcm^5^ modification on the corresponding tRNA^Arg^_UCU_. A group of 425 genes were identified with this unique codon usage and Trm9 was proposed to enhance their translation, which participates in an efficient response to DNA damage due to the fact that some of the targets are key players of that biological response.

**Figure 4:**
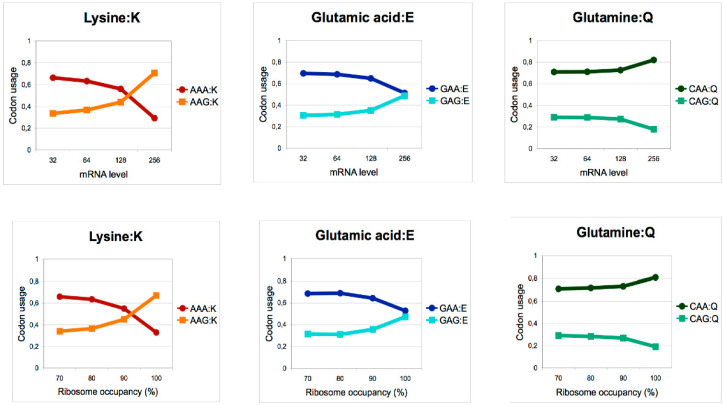
High protein expression in fission yeast is correlated with a skewed codon content excluding the AAA and GAA codons. Codon bias between G- and A-ending mcm^5^s^2^-modified codons for lysine, glutamic acid, and glutamine in genes as a function of mRNA level (upper panel) or ribosome occupancy (lower panel). Modified from Bauer et al., 2012 [37].

Inspired by the work of Begley, we later used a similar strategy in fission yeast to find that highly expressed genes have a skewed codon content, avoiding the AAA in favor of AAG, which is the alternative lysine codon (Figure 4). Remarkably, this occurs in the context of a G-C poor genome content (on average, 62% of lysine are encoded by AAA), suggesting that a selection pressure is operating to avoid the less efficient AAA-UUU codon-anticodon interaction when high expression is required [37]. We next analyzed the level of expression of the proteome using reverse protein arrays and reported that discrete functional groups of mRNAs were selectively affected [38], which was correlated to a skewed codon content for lysine. Taken together with the fact that the overexpression of the unmodified tRNA^LYS^_UUU_ was, by far, the most efficient suppressor of the phenotypes resulting from the inactivation of Elongator, the data supports the idea that the AAA codon is the most sensitive to the absence of modification. The translation of all other targeted anticodons is likely affected to various degrees. Key regulators of the cell cycle belonged to the target mRNAs, which may explain the cell cycle defect originally noted in a strain harboring the *sin3-193* (*elp3*) allele. In the case of one such target, *cdr2*, which encodes a repressor of the Wee1 kinase and a regulator of mitosis onset, the replacement of all AAA codons by the synonymous AAG completely uncoupled the level of expression of the protein from the presence of elongator [38].

The concept emerging from these works is that the co-regulated translation of groups of functionally connected mRNAs may result from their skewed codon content and dependency on a given tRNA modification pathway, namely the Elongator/Trm9 pathway in the present case [39]. Further works have supported these original findings, when it was shown that the translation of an increasing list of mRNAs can be uncoupled from the presence of Elongator by limiting the encoding of their lysine residues exclusively to the AAG codon. These mRNAs include *atf1*, which encodes a transcription factor that represents a downstream effector of the p38 MAP kinase pathway [40]. Similarly to the functional groups mentioned above, the main H_2_O_2_-dependent genes are highly expressed mRNAs containing a biased number of AAA lysine-coding codons versus AAG, thus making their mRNAs poorly translated after oxidative stress in cells lacking elongator [40].

In a totally different context, it was later reported that Elongator is required for Wnt-driven human intestinal tumor initiation by maintaining a pool of Lgr5(+)/Dclk1(+)/Sox9(+) cells. Mechanistically, elongator promotes Sox9 translation in a lysine AAA codon-dependent manner [41]. More recently, BRAF (V600E)-expressing human melanoma cells were shown to be dependent on Elongator for survival. Mechanistically, Elongator promotes glycolysis in melanoma cells through the direct, lysine AAA codon-dependent, regulation of the translation of HIF1A mRNA and the maintenance of high levels of HIF1α protein [42] (see below).

In addition, a study in mammals identified two categories of genes that require Elongator for normal expression and showed that genes in the DNA damage repair pathway are codon-biased, which couples them to the presence of Elongator in peripheral neurons [43]. In fission yeast, mRNAs encoding players of the TOR pathway, a conserved central regulator of growth, also have both a strong codon bias and a marked dependency to Elongator activity [44]. Therefore, they are now various examples in distantly related species and physiological contexts that Elongator participates in the coordinated expression of functionally related mRNA/proteins through their specific codon usage, especially for lysine.

## 3. Reprogramming of the mcm^5^ Modification by Cellular Signaling Controls Codon-Biased Translation in Various Contexts

If functionally related mRNAs are co-regulated due to their dependency to Elongator, the possibility of a regulation of this process through the control of Elongator activity is appealing. As indicated above, the fact that both a kinase and a phosphatase were recovered from the “zymocin screen” supports this hypothesis. 

**Figure 5:**
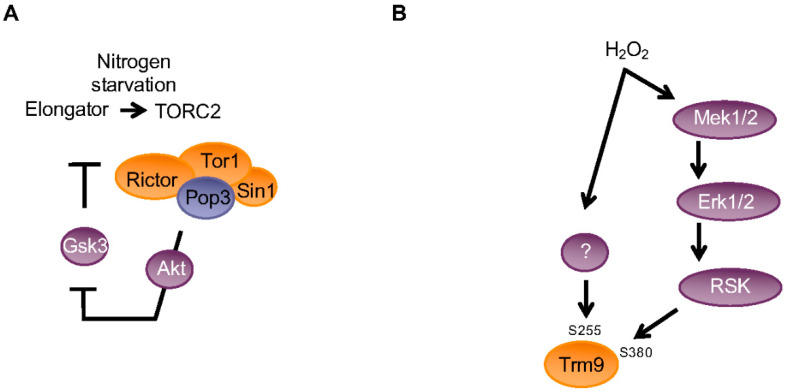
Schematic view of the integration of the Trm9 and Elongator-dependent mcm^5^ modification with cellular signaling pathways. (**A**) Elongator is a positive regulator of TORC2 in fission yeast by promoting the translation of Rictor and Tor1 components of the complex. In turn, TORC2 downregulates the activity of Gsk3, which is a negative regulator of Elongator through the phosphorylation of Elp4 on S114. This creates the positive feedback loop required to activate TORC2 and cell differentiation in nitrogen starvation conditions. (**B**) Phosphorylations of human TRM9L by various signalling cascades constitute a critical regulator of oxidative stress survival. Oxidative stress induces the rapid and dose-dependent phosphorylation of TRM9L on serine 380 downstream of the oxidative stress-activated MEK (mitogen-activated protein kinase kinase)-ERK (extracellular signal-regulated kinase)-RSK (ribosomal protein S6 kinase) signaling cascade. In addition, serine 255 phosphorylation is similarly responding to oxidative stress through an unknown pathway.

The concept of stress-specific reprogramming of modified ribonucleotides in tRNAs was developed by the team of Peter Dedon, first based on the Trm4 methylase [45]. In the proposed model, mRNAs characterized by a specific codon usage are more efficiently translated when the corresponding anticodons are modified. In addition, tRNA modifications can dynamically be regulated by stresses. The development and use of a bioanalytical pipeline [46] based on a sensitive liquid chromatography-tandem mass spectrometry (LC-MS/MS) to quantify a large spectrum of tRNA modifications revealed that this concept may expand far beyond a single modification and may be a new general principle of translational regulation, as developed in a previous review by Dedon, Begley, and colleagues [47]. For example, in response to alkylating agents, mcm^5^U and mcm^5^s^2^U were increased simultaneously to m^3^C and m^7^G. However, these marks were relatively unchanged in response to oxidizing agents, which suggests that a set of marks may be required to properly respond to a specific injury. Importantly, the codon/tRNA modification-based regulation may also be important in the absence of stress and integrated with the basal physiology of the cell. Notably, the levels of mcm^5^U and mcm^5^s^2^U fluctuate during the cell cycle, and mutations in Elongator result in cell cycle defects in fission yeast as indicated above, which supports this possibility. Our recent data demonstrated that the balance between the TORC1 and TORC2 complexes, which in fission yeast is critical as the decision to switch from proliferation to differentiation is partially regulated by Elongator through a translational control. In the event of starvation, when TORC2 is activated to induce differentiation, the Elongator-dependent tRNA modification is induced, which leads to reprogramming of translation and cell differentiation (Figure 5). Remarkably, the TORC2 complex also regulates the activity of Elongator by controlling a Gsk3-dependent phosphorylation of Elp4, generating a positive feedback loop [44]. 

Similarly, the Trm9 methylase was recently shown to integrate multiple stress-signaling pathways for tumor suppression in humans. Oxidative stress induces the rapid and dose-dependent phosphorylation of TRM9L downstream of the oxidative stress-activated MEK (mitogen-activated protein kinase kinase)-ERK (extracellular signal-regulated kinase)-RSK (ribosomal protein S6 kinase) signaling cascade [48]. 

Therefore, it is becoming clear that the activity of Elongator is controlled by cellular pathways and it will be very interesting to decipher all the molecular details of this integration, as well as its conservation in the future. In that context, the major recent advances in the understanding of the structure of Elongator provide the basis of a structure/function comprehension as to how Elongator activity is controlled. These aspects have been the topic of excellent previous reviews [49,50] and will be briefly discussed here. The team of Christoph Muller reported that the Elp4-5-6 subcomplex is an hexameric RecA-like ATPase that binds tRNAs in a manner regulated by ATP [51]. These findings constituted critical evidence that Elongator is a direct tRNA modification machinery. The architecture of the whole complex was revealed in 2017 [52,53] and recent works from the Sebastian Glatt laboratory have brought more insight about the catalytic activity of Elp3 and the molecular basis of tRNA recognition [54,55]. 

**Figure 6:**
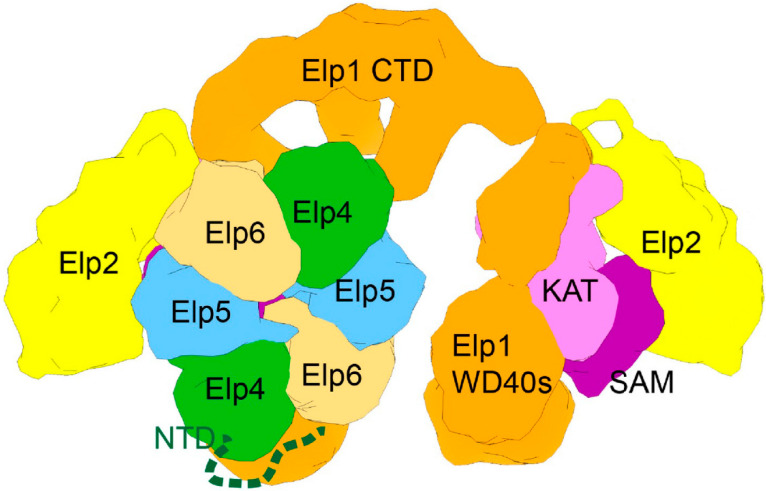
The remarkable asymmetrical structure of the Elongator complex. Elp1 (orange), Elp2 (yellow), Elp3 (pink), Elp4 (green), Elp5 (blue), and Elp6 (sand) are shown in a topological model of the two-lobed Elp1-2-3 subcomplex and asymmetrical binding of the hexameric RecA-like Elp4-5-6 ring. Individual domains of Elp1 (WD40, NTD and CTD) and the catalytic Elp3 subunit (KAT, SAM) are also indicated. Modified from Dauden et al., 2017 [52,53].

The most striking feature of the complex is its structural asymmetry. Two copies of Elp1, 2, and 3 arrange to form a two-lobe symmetric Elp1-2-3 subcomplex, where the two lobes are linked through dimerization of the Elp1 C-terminal domain. Elp1 forms a scaffold to which Elp2 binds at the periphery, while Elp3 is more central, performing tRNA modification at the interface of its HAT and SAM domains. Remarkably, the Elp4-5-6 ring binds to only one of the two lobes and both lobes get closer to each other to embrace the hexameric ring (Figure 6). Based on the decrease in tRNA affinity observed for Elp4-5-6 in the presence of ATP, it has been proposed that the RecA-like ring could be implicated in tRNA release from the Elp1-2-3 complex. Future works will reveal if a second Elp4-5-6 ring can bind the catalytic complex and if this is the basis of a regulation and a rate limiting step in the production of modified tRNAs. As already mentioned, the phosphorylation of Elp4 in fission yeast affects the level of Elongator-dependent tRNA modifications [44]. As indicated above, structural data support the idea that the role of the Elp4-5-6 subcomplex is related to active site clearance and efficient modified tRNA removal, rather than a direct contribution to the modification reaction. It will be interesting to test if the phosphorylation of Elp4 participates in that process and dynamically regulates it. Supporting a regulatory role of Elp4, genomic variants in human Elp4 have been associated with the appearance of centro-temporal spikes in Roland epilepsy, while microdeletions of the same Elongator subunit are associated with language impairment, autism spectrum disorder, and mental retardation [56,57]. 

As stated above, the dynamic regulation of Elongator was first suggested by the requirement of both the Kti14/Hrr25 kinase and the Sit4 phosphatase for sensitivity to zymocin. Pioneering work from Raffael Schaffrath explored this possibility in yeast and reported that these two enzymes affect the phosphorylation state of Elongator scaffold protein Elp1 on two sites, S1198 and S1202 [58,59,60]. However, though it is still unclear what precise role these phosphorylations play, it was proposed that increased or decreased phosphorylation could modulate the activity of Elongator by regulating the interaction of Elp1 with Hrr25/Kti14 and Kti12. In that context, it is interesting to note that dynamic phosphorylation of Elp1 was also observed in melanoma cells and changed in response to insulin availability [42].

Taken together, all these data indicate that Elongator may integrate the input from various cellular signaling pathways to reprogram translation in physiological or stress-induced conditions. The intriguing structure of the complex may have allowed this integration during the evolution of an ancestral monomeric Elp3 enzyme that still exists in some archaea. including *Methanocaldococcus infernus* [61] and bacteria including *Dehalococcoides mccartyi* [55] and whose main, constitutive function may be the maintenance of proteome integrity and suppression of proteins aggregation.

## 4. Elongator tRNA Modifications as a Protection from Protein Misfolding and Aggregation

The basis for the requirement of the Elongator-dependent tRNA modification has long been suspected to result from the low-stacking capacity of the unmodified AAA, GAA, and CAA codons, and the necessity to offset their translational inefficiency. This possibility was formally proven by the team of Sebastian Leidel in 2015 using ribosome profiling. The absence of mcm^5^s^2^U in yeast tRNA^GLU^_UUG_ and tRNA^LYS^_UUU_ was shown to result in a translational slowdown at cognate glutamine and lysine codons [62]. Variations in the rate of protein synthesis by ribosomes has previously been suggested to impact folding [63] and the Nedialkova and Leidel study demonstrated that codon-specific translation stalling in the absence of the mcm^5^s^2^U modification triggers the aggregation of proteins and threatens proteome homeostasis [62]. Interestingly, the same study highlighted accumulation of cellular protein aggregates largely overlapping those formed when the ribosome associated chaperone complexes (Ssb1/Ssb2) are compromised. These data also fit well with the fact that overexpression of the unmodified tRNA species can compensate for the lower codon-anticodon affinity and rescue the translation defect. 

However, in contrast to the coordinated codon-dependent regulation of translation detailed above, the aggregates observed when Elongator is absent are not enriched for proteins encoded by mRNAs with a skewed codon content. In addition, these aggregates are not abundant and their accumulation becomes much more obvious when both moieties of the mcm^5^s^2^ modification are absent in a double Elongator-thiolase mutant [62]. In addition, it should also be noted that the growth of an Elongator mutant is not significantly affected compared to an isogenic wild-type strain in the absence of stress. Altogether, these data may indicate that the aggregation protection role of Elongator may be a primary one, and maybe related to the ancestral role of Elongator resulting from a large-scale effect on all the Elongator-targeted anticodons that represent 25% of the tRNA population in budding yeast. A more specific and regulatory role of Elongator may have appeared during evolution by exploiting the intrinsic weakness of the AAA-UUU codon-anticodon pair. In that case, the activity of Elongator could be modulated, as seen for example in the case of the reciprocal regulation of the TOR pathway and Elongator [44], but maintained at a level avoiding global proteome-scale proteotoxic stress.

## 5. Elongator tRNA Modification and Diseases

Specific lesions affecting Elongator in humans result in a variety of distinct diseases, most commonly related to neurodegeneration, as recently reviewed in detail [64]. This may indicate that different Elongator subunits could be targeted by specific signaling cascades in specific cell type subpopulations, rather than a general proteotoxic effect that would be expected to result in a general much less specific phenotype [50].

The first link between Elongator and diseases was established when familial dysautonomia (FD) [65,66,67], a hereditary autonomic neuropathy, was shown to be caused by the mis-splicing of exon 20 in the *ELP1* gene, which results in a tRNA modification defect [68]. This causality was later demonstrated by the fact that treatment with RECTAS, a compound rectifying aberrant splicing, both increased the expression of Elp1 and recovered the Elongator-dependent tRNA modifications [69]. In addition, Elongator was more recently linked to the translation of codon-biased genes with both developmental and neurodegenerative phenotypes in FD [43].

Neurons are known to be particularly sensitive to translation defects [70] and while mutations affecting specific regulatory cascades may impact some cell populations, it is striking that protein aggregation is also a hallmark of several neurodegenerative diseases. Therefore, a less specific alteration of the Elongator complex may also participate in this type of diseases. A conditional knockout of *ELP3* in mice induced the unfolded protein response (UPR) associated with microcephaly [71]. More generally, the reduced translation efficiency resulting from the inactivation of elongator may cause protein aggregation in an unspecific manner, as defects in other modifications result in the same effects [72,73,74,75].

Remarkably, while the absence of Elongator mainly affects neurological processes, the teams of Alain Chariot and Pierre Close have accumulated evidence that this absence constitutes a major barrier to WNT-driven intestinal cancer initiation [41] and to metastasis of breast cancers [76]. In addition, the inactivation of Elongator strongly impedes the survival of melanoma cells to the primary treatment [42]. Indeed, it was shown that Elongator promotes glycolysis in melanoma cells through the XAA codon-dependent increase of HIF1α translation, which ultimately results in strong resistance to targeted therapy. Intriguingly, the upregulation of Elongator-dependent tRNA modification in that context was directly induced by the TORC2 pathway [42], which is reminiscent of the TOR/elongator co-regulated network highlighted in fission yeast [44], despite the fact that Elp1, rather than (or in addition to) Elp4, may be the phospho-target in that context. It will be interesting in the future to test if Elongator also positively regulates TORC2 during tumorigenesis.

## 6. Perspectives

The remarkable diversity of possible chemical modifications of tRNAs has been known for decades and much evidence has suggested that they are important to balance variations into translational efficiency and fidelity resulting from the biochemical properties of individual codon/anticodon combinations. The conservation of many of these modifications from pro- to eukaryotes supports this hypothesis. The cm5 chain synthetized by the Elongator complex as part of the double mcm^5^s^2^ modification found on three tRNAs is one of the most complex modifications, and recent evidence in various model systems support the idea that its synthesis requires a complex network of proteins interconnected with major cellular pathways. While the catalytic subunit Elp3 is necessary and sufficient to generate the modification in some archaea and bacteria, this enzyme associates in eukaryotes with 5 other conserved subunits to form a unique asymmetrical complex. An obvious hypothesis explaining this increased complexity is the positive selection and regulations of the complex during evolution, which seems to go hand in hand with a selection of strong codon usage bias to either avoid the Elongator-targeted anticodons, or conversely, to ensure the coordinated translation of sets of functionally related proteins. In addition, a less specific, maybe more ancestral role of Elongator in proteome maintenance also received large experimental support over the years. From a biomedical point of view, accumulating evidence suggests a critical role for Elongator in neurodevelopment, but also in tumorigenesis, indicating that these processes are both very dependent upon translation reprogramming. Detailed knowledge of how cellular signaling affects Elongator activity, the level of the modification, and the proteome will hopefully allow specific targeting by chemical compounds with therapeutic usage.
